# Continuity of care by a primary midwife (caseload midwifery) increases women’s satisfaction with antenatal, intrapartum and postpartum care: results from the COSMOS randomised controlled trial

**DOI:** 10.1186/s12884-016-0798-y

**Published:** 2016-02-03

**Authors:** Della A. Forster, Helen L. McLachlan, Mary-Ann Davey, Mary Anne Biro, Tanya Farrell, Lisa Gold, Maggie Flood, Touran Shafiei, Ulla Waldenström

**Affiliations:** Judith Lumley Centre, La Trobe University, 215 Franklin St., Melbourne, 3000 Australia; The Royal Women’s Hospital, Locked Bag 300, Cnr Grattan St and Flemington Rd, Parkville, 3052 Australia; School of Nursing and Midwifery, La Trobe University, Bundoora, 3086 Australia; School of Nursing and Midwifery, Monash University, Clayton, 3800 Australia; Deakin Health Economics, Deakin University, Burwood, 3125 Australia; Department of Women’s and Children’s Health, Division of Reproductive and Perinatal Health, Karolinska Institutet, Stockholm, Sweden

**Keywords:** Caseload midwifery, Continuity of care/r, Satisfaction, Experience, Randomised controlled trial

## Abstract

**Background:**

Continuity of care by a primary midwife during the antenatal, intrapartum and postpartum periods has been recommended in Australia and many hospitals have introduced a caseload midwifery model of care. The aim of this paper is to evaluate the effect of caseload midwifery on women’s satisfaction with care across the maternity continuum.

**Methods:**

Pregnant women at low risk of complications, booking for care at a tertiary hospital in Melbourne, Australia, were recruited to a randomised controlled trial between September 2007 and June 2010. Women were randomised to caseload midwifery or standard care. The caseload model included antenatal, intrapartum and postpartum care from a primary midwife with back-up provided by another known midwife when necessary. Women allocated to standard care received midwife-led care with varying levels of continuity, junior obstetric care, or community-based general practitioner care. Data for this paper were collected by background questionnaire prior to randomisation and a follow-up questionnaire sent at two months postpartum. The primary analysis was by intention to treat. A secondary analysis explored the effect of intrapartum continuity of carer on overall satisfaction rating.

**Results:**

Two thousand, three hundred fourteen women were randomised: 1,156 to caseload care and 1,158 to standard care. The response rate to the two month survey was 88 % in the caseload group and 74 % in the standard care group. Compared with standard care, caseload care was associated with higher overall ratings of satisfaction with antenatal care (OR 3.35; 95 % CI 2.79, 4.03), intrapartum care (OR 2.14; 95 % CI 1.78, 2.57), hospital postpartum care (OR 1.56, 95 % CI 1.32, 1.85) and home-based postpartum care (OR 3.19; 95 % CI 2.64, 3.85).

**Conclusion:**

For women at low risk of medical complications, caseload midwifery increases women’s satisfaction with antenatal, intrapartum and postpartum care.

**Trial registration:**

Australian New Zealand Clinical Trials Registry ACTRN012607000073404 (registration complete 23rd January 2007).

## Background

Continuity of carer has been strongly recommended and encouraged in maternity services in Australia [1], and many hospitals have responded by introducing caseload midwifery. In the caseload model women are cared for by a primary midwife throughout pregnancy, birth and the early postpartum period. We conducted a randomised controlled trial (RCT) comparing caseload midwifery care with standard care and found that women allocated to caseload midwifery were less likely to have a caesarean birth, analgesia during labour, and an episiotomy; that fewer infants were admitted to the special care nursery; and that mother and infant safety outcomes did not differ statistically between the study groups [2]. These findings confirm those of other RCTs of midwife-led care [3], suggesting that these models reduce interventions without jeopardising health outcomes.

Many of the RCTs of midwife-led care report increased satisfaction associated with being allocated to the midwife-led care trial arm [4–6]. However, a lack of consistency in measuring women’s satisfaction means that the Cochrane review *Midwife-led versus other models of care for childbearing women* was only able to report on satisfaction outcomes using a narrative approach [7]. While nine studies reported on maternal satisfaction, there was ambiguity around the concept of satisfaction, and inconsistency in the tools used to measure satisfaction [7]. Overall, although the included studies showed a higher level of satisfaction among women who had midwife-led care compared with those who did not, the reviewers could not identify which aspects of care increased women’s satisfaction. Other studies have reported associations between positive ratings of satisfaction and different aspects of care, such as conveniently located care [8], safe care with skilled professionals [8, 9], positive staff attitudes and behaviours (e.g. respectful, kind, empathetic) [8–11], being remembered between visits [12], having an active say in decision making [13], having enough information [8, 10, 13], perceiving care providers as helpful [13], and a consistent philosophy of care [11]. Both team midwifery models (that include four to 12 midwives) and caseload midwifery models (where women are allocated a primary ‘known’ midwife) have been shown to increase women’s satisfaction [4, 6, 14].

The type of continuity that matters to women has been extensively discussed; whether it is seeing the same care provider at each visit (continuity of carer) that is important, or if it is continuity in a broader sense, including the same team of midwives during all episodes of care, staff sharing the same philosophy of care, or consistency regarding guidelines, information and advice. The Cochrane review included ten trials in which midwife-led care was provided by teams, and only three trials of caseload midwifery, where there is a higher degree of continuity of carer [7]. The difference between team midwifery and caseload midwifery relates to the question of continuity of carer, and whether or not it is important to women.

A review by Green et al. [15] found no difference in satisfaction between women who had a known care provider during labour compared with those who had not. This review suggests that there is no evidence that women who are cared for in labour by a midwife they have already met are more satisfied than those who have not. The authors argue that other aspects of care, such as trust and consistent advice, may be more important to women than being cared for by a midwife with whom a relationship has developed over time. They also argue that although most of the schemes that aimed to achieve continuity of care did so, there should be caution in assuming that continuity is the specific component that explains the higher rates of satisfaction observed in the new models. Their concerns were based on the inconsistencies regarding the definitions of continuity; that studies evaluated packages of care rather than continuity as such; that while results showed that, as a group, women in models with higher continuity were more satisfied with their care, these results had not been analysed to examine whether the women who were more satisfied were also the ones who received higher levels of continuity; and finally, the potential bias caused by disappointment with the randomisation outcome in women allocated to standard care. A critical review by Freeman [16] found that the content of care was a higher priority for women than continuity of carer, and Waldenström found that continuity of carer was less important in a birth centre setting, where satisfaction was more likely to be associated with the attitudes of carers, the philosophy of care, and the environment, as opposed to knowing the individual midwife well [11]. In contrast to these findings, a summary of the literature by McCourt et al. [9] pointed to qualitative studies which all found continuity of carer to be important to women, particularly in labour.

Although evaluations of a ‘package’ of care such as caseload midwifery do not allow conclusions about which specific aspects of care contribute to the outcomes [2], secondary analyses of the data can contribute to the ongoing debate regarding the significance of continuity of carer, and whether or not it is associated with satisfaction.

The primary aim of this paper was to investigate the effect of caseload midwifery on women’s assessment of their satisfaction with antenatal, intrapartum, and postpartum care in hospital and at home. We investigated overall satisfaction with these episodes of care, as well as specific aspects of care, such as perception of emotional support, information and decision making, and whether care was provided in a competent way. A secondary aim of this paper was to explore the association between continuity of carer – being one of the key components of the caseload midwifery model – and satisfaction with care.

## Methods

### Study design and population

The study used a two-arm, randomised controlled design, stratified by parity (first or subsequent birth), to compare caseload midwifery care with standard maternity care [17]. The primary study aim was to explore the effect of caseload care on the percentage of women giving birth by caesarean section [2]. This paper addressed one of the secondary study aims – to explore the effect on women’s satisfaction with care [17]. Women were recruited from the Royal Women’s Hospital (the Women’s), a public tertiary women’s hospital in Melbourne, Australia, which has over 7,000 births per year. All eligible women booking to have a baby at the Women’s between September 2007 and June 2010 were approached to participate, except on occasions where no recruitment midwife was available (e.g. due to sick leave) or when all caseload midwifery places for the month had been already allocated. Inclusion criteria were: able to speak, read and write in English; singleton pregnancy; less than 24 completed weeks’ gestation and assessed as being at low obstetric risk at recruitment (more detail elsewhere) [17]. Women who had had a previous caesarean section were excluded. Caseload midwifery was not available to women outside the trial.

### Sample size

Sample size calculations for the trial were based on 80 % power to detect a reduction in the caesarean section rate from 19 to 14 % (*n* = 2,008) (with 95 % confidence). Given the rising caesarean rate, the data monitoring committee reviewed the sample size after two years of recruitment to check if the study remained adequately powered, and recommended an increase to 2290 (1145 women in each arm). In total 2,314 women were recruited. To detect a 10 % difference in the proportion of women satisfied with an overall episode of care (a pre-specified secondary outcome of the RCT) from (52 to 62 % or 52 to 42 %, using 52 % as a baseline estimate from our previous study in a similar population [6]) required 410 per group (with 80 % power and 95 % confidence); therefore the trial sample size was sufficient for this difference to be detected.

### Procedures

Women were recruited to the study by research midwives when attending their booking visit at the antenatal clinic, and randomised after written consent was obtained and the background questionnaire (collecting demographic data) completed. Randomisation was undertaken using an interactive voice response system activated by telephone (http://www.ctc.usyd.edu.au) using stratified permuted blocks of varying size [17].

### Caseload care

Women allocated to the intervention received the majority of their care from a ‘primary’ caseload midwife. If complications developed, the primary midwife collaborated with obstetricians and other health professionals and continued to provide caseload midwifery care. During pregnancy, women saw an obstetrician at the booking visit, 36 weeks and postdates (if required), and usually had one or two visits with a ‘back-up’ midwife. The primary midwife was on call for the woman’s labour and birth except in designated circumstances such as annual leave, sick leave, having already worked more than 12 h in a 24 h period, having more than one woman in labour, or if it was on one of the two days per week that the midwife was scheduled not to work or to be on call. Care was then provided by a back-up midwife, or on occasion, by non-caseload midwives. The primary midwife (or a back-up) attended the hospital on most days to provide some postpartum care and provided domiciliary care following discharge from hospital. All care was provided according to hospital guidelines and protocols. During the trial there were 10 (at commencement) to 14 midwives employed in caseload, equating to 7.5 to 12 full-time equivalent midwives. Midwives self-selected into the model. They were recruited from within the hospital and externally – and had to apply for a position. The only specific criterion was to have had two years experience post-registration, however other characteristics such as skills and midwifery philosophy were taken into account during the interview process.

### Standard care

For women allocated to standard care, options included midwifery-led care with varying levels of continuity, obstetric trainee care and community-based care ‘shared’ between a general medical practitioner (GP) and the Women’s, where the GP provided the majority of antenatal care. In the midwife- and GP-led models women saw an obstetrician at the booking visit, 36 weeks and postdates if required, with other referral or consultation as necessary. In all standard care options, women were cared for by whichever midwives and doctors were rostered for duty when they came into the hospital for antenatal, labour, birth and postpartum care. Care was provided according to the same hospital guidelines and protocols as for the women in caseload care.

### Intervention fidelity

At trial commencement, the caseload midwives attended information sessions emphasising the need to adhere to the Women’s clinical guidelines and to provide caseload care as defined in the study protocol. Adherence to intervention protocols was measured via interviews with caseload midwives at the beginning and end of the trial; regular meetings between caseload midwives and research team members; and data collected from the medical records. Intervention exposure measures included assessing the extent to which care was provided by the primary midwife (medical record data) and women’s recollection of having had a known care provider during pregnancy, labour, birth and the postpartum period (women’s survey data two months postpartum).

### Data collection

A postal questionnaire was mailed to all women two months after the birth, with the exception of those who had withdrawn, miscarried, had a perinatal death, or if either mother or infant had a serious medical problem. Reminder letters were sent to non-responders two and four weeks after the initial mail out [17]. The questionnaire was largely based on previous studies of models of care conducted in Victoria [6, 18]. Likert-type scales (where ‘1’ signified ‘disagree strongly’ and ‘7’ signified ‘agree strongly’) were used for a range of specific and global questions regarding women’s satisfaction with antenatal, intrapartum and postpartum care. Women were also asked about the presence of known care providers for labour, birth, postpartum hospital care and domiciliary care.

### Measures of continuity

Medical record data were obtained to describe intervention exposure. All other continuity variables (e.g. women’s recollection of having previously met the midwife caring for her in labour) were obtained from the two month questionnaire (i.e. self-reported). Continuity in labour and birth was measured by the number of midwives who looked after the woman during labour and birth, and by the number of times a woman had met the best-known of these midwives before. Given that many women had a number of midwives caring for them in labour, it was decided that the midwife they had ‘met most often before labour’ would be used to quantify a ‘dose’ of known care provider. This dose variable was explored in single increments up to having met a midwife six times previously, then the remainder grouped as having previously met a midwife ‘seven times or more’. A further question on labour care was included – “Would you have liked to get to know the midwife attending the birth better before you had the baby?” Continuity was not explored for in-hospital postnatal care, but women were asked whether they had previously met any of the midwives providing postnatal care at home (domiciliary care).

### Data analysis

STATA 10.0 and 11.2 were used for all data analysis [19, 20]. The primary analyses were by intention to treat, that is, data were analysed by randomised group regardless of care received or of any protocol deviations [21]. Where women were asked to rate their care by responding to statements on a seven-point scale, responses were compared by trial arm using the ‘ologit’ command in STATA to undertake ordinal logistic regression (to make use of all the data on the scale). The results are presented as proportional odds ratios (ORs) and 95 % confidence intervals (CIs). Comparison of means was undertaken for continuous variables, using t-tests where data were normally distributed; otherwise medians were compared using Mann–Whitney U tests. In the secondary analyses of satisfaction with labour and birth care related to continuity of carer in labour, binary logistic regression was used, and ORs 95 % CIs presented.

Ethics approval was obtained from the Royal Women’s Hospital (Project 07/01) and La Trobe University Human Research Ethics Committees (Project 07/04). The trial is registered with the Australian New Zealand Clinical Trials Registry (ACTRN012607000073404).

## Results

### Trial participants

**Fig. 1:**
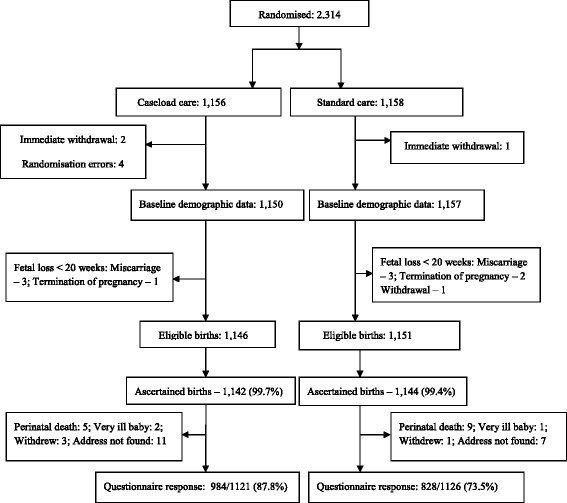
Trial profile

Of the 2,314 women recruited to the study, 1,156 were allocated to caseload midwifery and 1,158 to standard care, resulting in 1,146 and 1,151 eligible women in the respective groups (Fig. 1). Full trial profile data has been published elsewhere [2]. Response fractions for the two-month questionnaire were 87.8 % (984/1121) in the caseload care group and 73.5 % (828/1126) in the standard care group.

Of those responders to the postnatal follow-up who had been allocated to caseload care, 16 received other forms of care: three at the Women’s in standard care models, and the rest elsewhere. Of those allocated to standard care, 76 % (632/828) had public antenatal care with midwives, 2 % (17/828) obstetric trainee care; 17 % (172/828) shared care with a family doctor (GP); and 5 % (38/828) transferred their care from the Women’s (at varying times during pregnancy) to other hospitals or alternative models such as birth centre care or home birth. In both trial arms, women had additional specialist obstetric care as required, and in both, a few women birthed elsewhere after having all their care at the Women’s (e.g. preterm births while away from home). These data are similar to the overall COSMOS sample [2].

### Intervention exposure

Women allocated to caseload midwifery who responded to the two-month questionnaire had a mean of 4.2 (SD 1.4) pregnancy visits with their primary midwife and 1.9 (SD 1.2) with a back-up midwife. During pregnancy, 99.7 % (977/980) saw their primary midwife at least once and 86.0 % (843/980) had at least one visit with a back-up midwife. During labour and birth, the primary midwife provided care for 58.4 % (573/981) of the respondents allocated to caseload care, and the back-up midwife for 49.0 % (481/981) of the women. Overall, 90.6 % of respondents allocated to caseload care were cared for in labour by either their primary and/or back-up midwife. Reasons women did not receive any care from their primary or back-up midwife during labour and birth included the midwife not being called or not being called in time, or that neither the caseload midwife nor her back-up midwife were available (e.g. if both had already worked the total hours permitted). A small number of women transferred care away from the hospital. In the postnatal period, 94.4 % (926/981) of respondents in the caseload group received some care in hospital by their primary and/or back up midwife. This included one to two hours of postnatal care per day from the caseload midwife, with core staff providing other care as required. Most (92.8 %; 910/981) received postnatal domiciliary care by their primary and/or back up midwife. Again, these data are similar to those reported for the overall COSMOS sample [2].

### Respondents at two months postpartum

Of those who responded at two months, background characteristics were similar between the trial arms (Table 1). Compared with the characteristics of the overall sample in the COSMOS trial, responders to the postal survey were slightly less likely to have a low family income (caseload 8.3 % of women responding to the two-month to the survey vs 10.8 % of the sample overall; standard care 7.6 % of women responding to the two-month to the survey vs 12.1 % of the sample overall); and to be receiving government benefits as the main family income (caseload 2.4 % vs 3.7 %; standard care 3.0 % vs 5.9 %). Responders to the survey were also slightly more likely to be born in Australia (caseload 60.4 % vs 58.4 %; standard care 63.0 % vs 57.7 %) and to have English as a first language (caseload 79.8 % vs 78.0; standard care 82.3 % vs 78.1 %) than those in the overall sample.

**Table 1:** Participant characteristics of women responding to questionnaire two months postpartum

	Caseload		Standard care	
	*n* = 984		*n* = 828	
	n	%	n	%
Age at booking visit, mean (sd)	31.5	4.4	32.0	4.6
Gestation at booking, mean (sd)	16.2	2.7	16.2	2.9
Expecting first baby	696	70.6	572	69.1
Married/living with partner (972/812)^a^	929	95.6	774	95.3
Highest education level (971/808)^a^				
Completed degree/diploma	769	79.2	627	77.6
Completed secondary school	151	15.6	134	16.6
Did not complete secondary school	51	5.3	47	5.8
Total family income/year (AUD)				
< $33,800 per year	82	8.3	63	7.6
$33,801 to 51,999 per year	164	16.7	107	12.9
$52,000 to 72,799 per year	194	19.7	169	20.4
$72.800-103,999 per year	274	27.9	234	28.3
$104,000 or more per year	263	26.7	241	29.1
Pension/benefit main family income	24	2.4	25	3.0
Smoked prior to pregnancy	164	16.7	143	17.5
Born in Australia (956/797)^a^	577	60.4	502	63.0
English first language	892	79.8	681	82.3

^a^Numbers in parentheses indicate number for whom this information was available (Caseload/Standard care)

### Satisfaction with care

Caseload midwifery was associated with more favourable ratings of satisfaction with care across all care episodes. The strongest effects were in pregnancy care and postpartum care at home.

#### Care during pregnancy

There was no difference in the total number of antenatal visits reported by women in the caseload (mean 9.0 visits, SD 3.4) or standard care (mean 9.0 visits, SD 8.5) groups (*p* = 0.10), however women in caseload care saw fewer different midwives (mean 2.8 midwives, SD 1.2) than those in standard care (mean 4.6 midwives, SD 2.6) (*p* < 0.001) (Table 2).

**Table 2:** Self-reported continuity of carer

Aspect of care	Caseload		Standard care		Test statistic
Pregnancy					
Total number of antenatal visits (mean, SD) (953/790)	9.0	3.4	9.0	8.5	Mean diff 0.26, *p* = 0.10
Number of different midwives seen during pregnancy (mean, SD, (range)) (960/794)	2.8	1.2 (0, 14)	4.6	2.6 (0, 30)	Mean diff 1.82, *p* < 0.001
Number of midwives who provided care in labour (mean, SD, (range)) (977/820)	2.4	1.3 (0, 16)	3.3	2.1 (0, 18)	Mean diff 0.91, *p* < 0.001
Labour and birth					
Number of times had met the ‘best known’ intrapartum midwife (mean, SD, (range)) (966/820)	3.7	3.0 (0, 20)	0.2	0.3 (0, 10)	Mean diff 3.46, *p* < 0.001
Median (IQR (median included here as this data more skewed)	3	(1, 6)	0	(0, 0)	*p* < 0.001
					(Mann–Whitney test)
Had met at least one of midwives providing care in labour at least once before (n, %) (966/820)	853	88.3	74	9.0	Chi^2^ *p* < 0.001
Number of times had previously met at least one of the midwives providing care in labour (n, %) (966/820):					Chi^2^ *p* < 0.001
Had met none of midwives	113	11.7	746	91.0	
Once	198	20.5	45	5.5	
Two times	100	10.4	12	1.5	
Three times	91	9.4	4	0.5	
Four times	112	11.6	2	0.2	
Five times	105	10.9	4	0.5	
Six times	98	10.1	2	0.2	
Seven times or more	149	15.4	5	0.6	
Would have liked to get to know midwife attending birth better before had baby (n, %) (917/818)					Chi^2^ *p* < 0.001
Yes, definitely	331	36.1	405	49.5	
Yes, possibly	235	25.6	247	30.2	
No, not really	351	38.3	166	20.3	
Postpartum					
Had a least one postnatal visit at home with midwife met before (n, %) (957/777)	896	96.3	123	15.8	Chi^2^ *p* < 0.001

When asked an overall global question about their care during pregnancy, women in the caseload group were over three times more satisfied than women in standard care (OR 3.35, 95 % CI 2.79, 4.03; *p* < 0.001) (Table 3). Compared with women in standard care, women in the caseload group were more likely to report that they were asked if they had any questions; that midwives kept them informed; that they were given an active say about decisions; that their worries, anxieties or concerns were taken seriously; that reassurance was given by midwives when needed; that midwives were less often rushed; and that care was provided safely and competently. Women in caseload care also reported that they were happier with the physical and emotional support provided by midwives, but were less satisfied with care provided by doctors than women in standard care.

**Table 3:** Satisfaction with pregnancy care

	Satisfaction scores (%)			Caseload care *Standard care* ^b^				OR^a^	95 % CI	P
	1	2	3	4	5	6	7			
	Disagree strongly						Agree strongly			
At my check-ups I was always asked whether I had any questions (982/*827*)	0.0	0.1	0.2	1.3	3.2	13.8	81.5	2.58	2.08, 3.19	<0.001
	*1.0*	*1.2*	*2.1*	*3.3*	*8.8*	*19.4*	*64.3*			
The midwives always kept me informed about what was happening (982/*821*)	0.1	0.2	0.5	1.5	5.1	19.9	72.7	4.29	3.54, 5.19	<0.001
	*1.6*	*1.3*	*3.4*	*6.6*	*17.7*	*29.4*	*40.1*			
The doctors always kept me informed about what was happening (946/*819*)	2.6	3.5	7.7	13.7	18.5	22.8	31.1	0.93	0.79, 1.10	0.40
	*2.3*	*3.7*	*6.1*	*11.6*	*20.8*	*24.7*	*30.9*			
I was always given an active say in decisions about my care in pregnancy (982/*820*)	0.3	0.4	0.7	3.7	7.8	26.2	60.9	3.18	2.66, 3.81	<0.001
	*1.1*	*2.1*	*4.3*	*10.2*	*19.2*	*27.8*	*35.4*			
I always felt my worries, anxieties or concerns about the pregnancy and the baby were taken seriously by the midwives (978/*816*)	0.8	0.3	0.7	1.7	4.4	15.8	76.3	4.04	3.33, 4.93	<0.001
	*1.6*	*1.7*	*2.9*	*6.9*	*12.0*	*30.9*	*44.0*			
I always felt my worries, anxieties or concerns about the pregnancy and the baby were taken seriously by the doctors (925/*816*)	2.7	2.9	7.0	13.6	17.6	25.7	30.4	0.84	0.71, 0.99	0.04
	*2.1*	*3.7*	*5.5*	*11.3*	*16.4*	*27.3*	*33.7*			
The midwives provided reassurance when I needed it (979/*813*)	0.2	0.3	0.7	1.7	5.1	17.3	74.7	3.89	3.21, 4.71	<0.001
	*0.9*	*1.2*	*2.5*	*8.7*	*16.0*	*29.4*	*41.3*			
The doctors provided reassurance when I needed it (920/*811*)	3.6	5.3	5.9	15.3	21.1	22.2	26.6	0.78	0.66, 0.92	0.003
	*2.3*	*3.7*	*5.6*	*13.1*	*18.0*	*28.4*	*29.0*			
At my check-ups the midwives often seemed rushed (982/*821*)	54.9	25.6	7.2	4.9	3.8	2.3	1.3	0.19	0.16, 0.23	<0.001
	*20.2*	*21.7*	*14.0*	*15.0*	*12.8*	*8.2*	*8.2*			
At my check-ups the doctors often seemed rushed (944/*819*)	13.1	14.0	9.8	15.2	15.9	17.0	15.2	1.11	0.94, 1.31	0.21
	*12.6*	*16.0*	*12.3*	*13.3*	*16.4*	*16.4*	*13.1*			
Care in pregnancy was provided in a competent way (969/*818*)	0.1	0.3	0.9	2.5	7.5	26.7	61.9	3.09	2.57, 3.70	<0.001
	*0.5*	*1.1*	*2.8*	*8.7*	*16.4*	*35.3*	*35.2*			
I was happy with the emotional support I received in pregnancy from midwives (977/*812*)	0.4	0.4	0.6	1.6	4.4	18.6	73.9	5.02	4.13, 6.19	<0.001
	*1.6*	*1.6*	*2.1*	*12.1*	*15.4*	*29.9*	*37.3*			
I was happy with the emotional support I received in pregnancy from doctors (907/*809*)	7.5	6.7	9.2	22.5	20.2	17.2	16.8	0.62	0.52, 0.73	0.001
	*3.8*	*3.7*	*6.7*	*21.3*	*18.3*	*23.9*	*22.4*			
I was happy with the physical care I received in pregnancy from midwives (977/*818*)	0.0	0.3	0.5	1.2	4.4	20.0	73.6	3.91	3.23, 4.75	<0.001
	*0.7*	*0.4*	*2.0*	*6.7*	*11.7*	*36.7*	*41.8*			
I was happy with the physical care I received in pregnancy from doctors (925/*818*)	2.3	3.6	5.1	15.4	16.9	25.1	31.8	1.26	1.46, 1.49	0.008
	*2.1*	*2.2*	*3.8*	*9.5*	*16.8*	*34.0*	*31.7*			
Overall, how would you describe your care during pregnancy	0.1	0.2	0.3	1.1	6.8	29.9	61.6	3.35	2.79, 4.03	<0.001
(1 = very poor; 7 = very good) (976/*823*)	*0.6*	*0.7*	*2.6*	*7.2*	*16.7*	*38.0*	*34.3*			

^a^OR is proportional odds ratio derived from ordinal logistic regression

^b^Italicised figures on second rows are Standard Care results

#### Intrapartum care

Women in the caseload arm reported having fewer midwives on average caring for them during labour and birth (mean 2.4 midwives, SD 1.3) than those in standard care (mean 3.3 midwives, SD 2.1) (*p* < 0.001) (Table 2). They also reported having met the ‘best known’ midwife providing labour and birth care more often during the antenatal period than those women allocated to standard care (median 3 and 0 respectively, *p* < 0.001). A total of 88.3 % (853/966) of women in caseload care reported having previously met at least one of the midwives caring for them in labour and birth at least once, compared with 9.0 % (74/820) of women in standard care.

Women randomised to caseload midwifery were more satisfied with all midwife-related measures of intrapartum care than women in standard care (Table 4). They more often felt they had an active say in decisions about care during labour and birth; that their privacy needs were met; that midwives were encouraging, reassuring and emotionally supportive and that care was provided safely and competently. Overall, women in caseload care were twice as satisfied with care during labour and birth compared with women in standard care (OR 2.13, 95 % CI 1.78, 2.56; *p* < 0.001).

**Table 4:** Satisfaction with care during labour and birth

	Satisfaction scores (%)			Caseload care *Standard care* ^b^				OR^a^	95 % CI	P
	1	2	3	4	5	6	7			
	Disagree strongly						Agree strongly			
The midwives always kept me informed about what was happening (973/*821*)	0.5	1.0	1.0	3.7	11.3	23.3	59.1	2.23	1.87, 2.66	<0.001
	*1.6*	*2.8*	*5.0*	*5.9*	*14.6*	*31.2*	*39.0*			
The doctors always kept me informed about what was happening (716/*670*)	*3.5*	*4.1*	*5.0*	*11.6*	*18.0*	*21.9*	*35.9*	1.09	0.90, 1.32	0.36
	*3.0*	*4.8*	*6.4*	*11.3*	*16.4*	*26.0*	*32.1*			
I was always given an active say in decisions about my care during labour and birth (964/*817*)	1.0	1.7	3.1	6.3	13.4	26.4	48.1	1.91	1.61, 2.26	<0.001
	*2.8*	*3.9*	*5.1*	*11.3*	*16.3*	*27.8*	*32.8*			
The midwives were encouraging (972/*818*)	0.1	0.3	0.8	1.7	4.2	16.0	77.0	2.82	2.31, 3.44	<0.001
	*0.6*	*1.5*	*2.9*	*4.4*	*9.3*	*26.7*	*54.7*			
The doctors were encouraging (699/*656*)	4.7	4.2	5.3	15.3	16.0	20.3	34.2	0.99	0.82, 1.2	0.94
	*3.7*	*6.0*	*4.7*	*12.0*	*18.3*	*22.9*	*32.3*			
The midwives provided reassurance if I needed it (970/*819*)	0.3	0.6	0.3	2.3	6.0	16.9	73.6	2.62	2.12, 3.17	<0.001
	*1.0*	*1.6*	*2.2*	*6.1*	*10.3*	*27.1*	*51.8*			
The doctors provided reassurance if I needed it (698/*660*)	5.6	4.4	5.3	15.9	19.2	21.4	28.2	0.88	0.73, 1.07	0.20
	*4.1*	*5.2*	*5.5*	*12.4*	*20.3*	*22.6*	*30.0*			
Care during labour and birth was provided in a safe way (973/*823*)	0.6	0.9	1.2	1.3	6.4	17.9	71.6	1.73	1.43, 2.10	<0.001
	*1.0*	*0.9*	*1.5*	*4.0*	*9.1*	*24.4*	*59.2*			
Care during labour and birth was provided in a competent way (968/*818*)	1.2	0.9	1.0	2.6	6.5	18.6	69.1	1.84	1.52, 2.21	<0.001
	*1.2*	*1.0*	*2.6*	*5.0*	*10.3*	*25.3*	*54.7*			
I was happy with the emotional support I received from midwives (975/*815*)	0.7	0.5	0.9	1.7	5.4	17.0	73.6	2.71	2.23, 3.29	<0.001
	*1.7*	*1.2*	*4.2*	*5.2*	*10.9*	*25.4*	*51.4*			
I was happy with the emotional support I received from doctors (700/*664*)	7.6	3.9	5.1	18.6	17.0	20.9	27.0	0.89	0.74, 1.07	0.22
	*5.7*	*6.0*	*6.2*	*12.7*	*17.9*	*22.6*	*28.9*			
My privacy needs were well respected during labour and birth (964/*814*)	0.8	0.5	2.0	6.4	6.9	17.7	65.7	1.68	1.39, 2.01	<0.001
	*1.5*	*2.5*	*1.8*	*6.8*	*10.0*	*25.4*	*52.1*			
Overall, how would you describe your care in labour and birth? (1 = very poor; 7 = very good) (976/*813*)	0.7	0.6	1.2	2.1	7.3	22.9	65.3	2.13	1.78, 2.56	<0.001
	*0.7*	*1.5*	*2.5*	*5.3*	*12.8*	*30.4*	*46.9*			

^a^OR is proportional odds ratio derived from ordinal logistic regression

^b^Italicised figures on second rows are Standard Care results

#### Postpartum care

Although the caseload model was not tied to length of stay, women allocated to the caseload care arm in the original sample stayed less time in hospital postpartum that those allocated to standard care on average (55.4 h [SD 0.97] vs 60.5 h [SD 0.78]; *p* < 0.001) [2]. Those who responded to the survey were similar; more women allocated to caseload care compared with those in standard care left hospital within 24 h of the birth (7.5 vs 3.5 %; *p* < 0.001); and within 48 h of the birth (39.7 vs 26.9; *p* = <0.001).

Compared to women in standard care, women in the caseload group reported higher satisfaction with postnatal care overall (OR 1.56, 95 % CI 1.32, 1.85; *p* < 0.001) and were more likely to report feeling informed by midwives; having had an active say in decisions about care of themselves and their baby; that midwives were sensitive, encouraging and emotionally supportive; that midwives were not rushed; and that care was provided safely and competently (Table 5). They were also more likely to report that they were given the advice they needed with breastfeeding, handling, settling and caring for the baby and about their own health and recovery after the birth.

**Table 5:** Satisfaction with postpartum care in hospital (bottom row – rating of care at home)

	Satisfaction scores (%)			Caseload care *Standard care* ^b^				OR^a^	95 % CI	P
	1	2	3	4	5	6	7			
	Disagree strongly						Agree strongly			
The midwives always kept me informed about what was happening (969/*820*)	0.7	2.6	5.4	8.3	13.8	27.7	41.6	1.95	1.65, 2.31	< 0.001
	*3.1*	*4.2*	*10.0*	*9.8*	*20.0*	*26.0*	*27.1*			
The doctors always kept me informed about what was happening (856/*755*)	6.7	6.2	9.2	19.2	15.8	19.4	23.6	1.01	0.85, 1.19	0.06
	*7.3*	*7.8*	*9.7*	*13.1*	*18.5*	*20.8*	*22.8*			
I was always given an active say in decisions about care of my baby and myself (970/*815*)	1.4	1.7	3.7	8.8	14.3	26.2	43.9	1.62	1.37, 1.92	< 0.001
	*2.5*	*4.5*	*5.9*	*10.6*	*17.3*	*26.1*	*33.1*			
I was given the advice I needed with breast feeding (967/*812*)	2.0	3.1	4.7	7.9	13.2	24.9	44.3	1.60	1.35, 1.89	< 0.001
	*5.5*	*5.4*	*7.3*	*8.6*	*15.3*	*22.7*	*35.2*			
I was given the advice I needed about how to handle, settle or look after my baby (962/*808*)	3.9	5.6	7.8	15.4	18.6	19.3	29.4	1.50	1.27, 1.77	< 0.001
	*6.9*	*8.4*	*9.4*	*14.5*	*22.4*	*17.0*	*21.4*			
I was given the advice I needed about any problems with the baby’s health and progress (947/*806*)	1.2	1.7	4.7	11.1	15.6	27.4	38.4	1.75	1.48, 2.08	< 0.001
	*4.3*	*4.7*	*5.7*	*13.3*	*19.5*	*25.4*	*27.1*			
I was given the advice I needed about my own health and recovery after the birth (972/*820*)	2.1	1.3	3.7	8.6	13.8	28.7	41.8	1.80	1.52, 2.13	< 0.001
	3.7	4.5	6.3	11.3	18.4	25.6	30.1			
The midwives were sensitive (962/816)	1.7	2.0	2.4	9.8	13.7	24.5	46.0	2.09	1.76, 2.48	< 0.001
	*2.8*	*3.7*	*5.6*	*11.2*	*21.6*	*29.0*	*26.1*			
The doctors were sensitive (865/*760*)	4.7	4.2	7.3	22.9	18.2	19.5	23.2	1.08	0.91, 1.29	0.36
	*5.0*	*4.3*	*6.8*	*21.3*	*21.8*	*22.9*	*17.8*			
The midwives were encouraging (971/*817*)	0.4	1.3	2.7	5.7	13.9	25.8	50.3	2.04	1.71, 2.42	< 0.001
	*1.5*	*2.7*	*4.2*	*10.7*	*18.6*	*30.0*	*32.4*			
The doctors were encouraging (859/*756*)	*4.3*	*4.8*	*6.8*	*20.8*	*18.5*	*20.7*	*24.1*	1.06	0.89, 1.26	0.52
	*4.1*	*5.2*	*5.4*	*21.4*	*20.2*	*23.8*	*19.8*			
The midwives often seemed rushed (968/*820*)	25.6	18.4	14.1	10.7	11.3	12.2	7.8	0.32	0.27, 0.37	< 0.001
	*9.5*	*11.5*	*8.2*	*13.2*	*15.1*	*18.5*	*24.0*			
The doctors often seemed rushed (873/*759*)	13.3	10.3	11.0	21.0	15.1	16.4	12.9	0.69	0.58, 0.82	< 0.001
	*7.5*	*8.6*	*9.9*	*21.0*	*19.0*	*15.6*	*18.6*			
Care in hospital after the birth was provided in a competent way (967/*815*)	1.7	1.5	3.8	8.8	17.0	27.1	40.2	1.36	1.15, 1.61	< 0.001
	*2.7*	*3.3*	*4.1*	*10.6*	*17.1*	*30.1*	*32.3*			
I was happy with the emotional aspects of care by midwives (967/*811*)	1.6	2.1	2.9	9.6	13.4	23.7	46.7	2.05	1.73, 2.43	< 0.001
	*3.6*	*4.4*	*5.8*	*11.5*	*19.0*	*27.5*	*28.2*			
I was happy with the emotional aspects of care by doctors (857/*747*)	4.8	6.0	8.5	25.3	19.5	15.8	20.2	0.95	0.80, 1.13	0.53
	*5.8*	*5.6*	*7.4*	*22.1*	*21.3*	*19.3*	*18.6*			
Overall, how would you describe the care you received in hospital after the birth? (1 = very poor; 7 = very good) (970/*813*)	1.7	2.3	4.4	8.3	16.3	30.7	36.4	1.56	1.32, 1.85	< 0.001
	*3.4*	*3.4*	*7.5*	*10.6*	*19.1*	*27.7*	*28.3*			
Overall, how would you describe the care your baby received in hospital after the birth? (1 = very poor; 7 = very good) (969/*814*)	1.0	1.1	2.4	6.3	14.0	28.9	46.2	1.30	1.10–1.55	0.002
	*1.6*	*1.8*	*3.9*	*6.4*	*15.0*	*32.1*	*39.2*			
Care at home	1	2	3	4	5	6	7			
	Very poor						Very good			
Overall, how would you describe the care you and your baby received from hospital staff at home after the birth? (957/768)	0.5	0.4	1.2	2.3	8.6	18.9	68.1	3.19	2.64, 3.85	< 0.001
	1.3	2.7	3.8	10.8	13.7	26.7	41.0			

^a^OR is proportional odds ratio derived from ordinal logistic regression

^b^Italicised figures on second rows are Standard Care results

#### Postpartum care at home

Women in caseload care reported more postnatal midwife visits at home than women in standard care (mean 2.5 visits, SD 0.04, compared with 1.8 visits, SD 0.03; *p* < 0.001; *n* = 948/760), and were more likely to have previously met one of the midwives who visited them (96.3 % vs. 15.8 %, *p* < 0.001) (Table 2). In response to an overall question about care provision for mother and baby at home after the birth, women randomised to caseload were three times more satisfied than women in standard care (OR 3.19, 95 % CI 2.64, 3.85; *p* < 0.001) (Table 5). This may be explained in part by the higher number of home-based postnatal visits.

### Further exploration of the findings

**Fig. 2:**
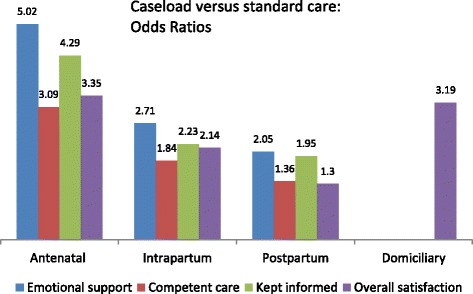
Comparison of women’s assessment of different aspects of antenatal, intrapartum and postpartum care, and overall assessment of these episodes of care and domiciliary postnatal care. Odds Ratios (ORs) based on comparisons of score on 7-point scales ranging from ‘1’ (Disagree strongly) to ‘7’ (Agree strongly). All p values for ORs shown are *p* < 0.001

Figure 2 provides a visual presentation of the ORs for selected items from Tables 3, 4 and 5 where satisfaction was much higher in the caseload group than in the standard care group (*p* < 0.001), and where the items illustrate different aspects of care. We selected one item from each of the following aspects: emotional aspects of care, information and decision making, and competent care/physical care, and have included the overall satisfaction OR for each component of care. The figure shows that the difference between the women’s assessments in the caseload and standard care groups was most pronounced regarding emotional support, and this was most obvious in the assessment of pregnancy care (OR 5.02; 95 % CI 4.13, 6.19). The odds of being kept informed by the midwives about what was happening during each of the respective episodes of care was also increased in women in the caseload group, again, most during pregnancy (OR 4.29; 95 % CI 3.54, 5.19). Similarly, women in caseload were more likely to consider their care was provided in a competent manner (pregnancy OR 3.09; 95 % CI 2.54, 3.70) and to rate their overall satisfaction higher (pregnancy OR 3.35; 95 % CI 2.79, 4.03).

#### Known care provider in labour and overall satisfaction with intrapartum care

Table 2 shows the comparative amounts of continuity of carer in the two trial arms. To look further at any association between having a known care provider in labour and the effect on satisfaction with labour care, the trial arms are looked at separately. In these analyses the ORs refer to the comparison of women scoring ‘0’ to ‘5’ *versus* ‘6’ or ‘7’ on the scale for overall satisfaction with labour and birth care, where ‘7’ indicates the highest satisfaction.

Due to the small numbers of women in the standard care group who had previously met a midwife before labour, the comparison in this group was restricted to having met at least one of the midwives compared with not. No difference in the rating of overall labour and birth satisfaction was found between women in the standard care group who had previously met a midwife who cared for them in labour compared with those who had not (OR 1.07, 95 % CI 0.60, 1.91; *p* = 0.82).

In the caseload group, if women had previously met at least one of midwives providing care in labour at least once before, they were more likely to be satisfied with their labour care overall (OR 1.83, 95 % CI 1.07, 3.12; *p* = 0.03). Table 6 shows that satisfaction with intrapartum care in the caseload group increased by the number of times the woman had met the midwife before, up to four times. Only the difference at four times was statistically significant (OR 3.38, 95 % CI 1.36, 8.34).

**Table 6:** Association between knowing a midwife providing labour and birth care and overall satisfaction with labour and birth care (caseload group only). ORs based on comparison of women scoring ‘0’ to ‘5’ *versus* ‘6’ or ‘7’ where ‘7’ indicates the highest satisfaction

	Number scoring ‘6’ or ‘7’	%	OR	95 % CI	p-value
Had met at least one of the midwives providing care in labour at least once before					
No	89/109	81.7	1	(ref)	
Yes	756/849	89.1	1.83	1.07, 3.12	0.03
Number of times had previously met at least one of the midwives providing care in labour (*n* = 958)					
Never	89/109	81.7	1	(ref)	
Once	169/197	85.8	1.36	0.72, 2.54	0.34
Twice	89/99	89.9	2.00	0.89, 4.51	0.10
Three times	82/91	90.1	2.05	0.89, 4.75	0.10
Four times	105/112	93.8	3.38	1.36, 8.34	0.009
Five times	92/105	87.6	1.59	0.74, 3.39	0.23
Six times	88/97	90.7	2.12	0.94, 5.10	0.07
Seven times or more	131/148	88.5	1.73	0.86, 3.49	0.12

As shown in Table 2, women in caseload care were less likely than those in standard care to express a desire to know their midwife better, although even for women in caseload who had met one of the midwives who cared for them in labour four times or more, 34.6 % (145/419) still ‘definitely’ would have liked to know the midwife at the birth better. In both groups women who said they ‘definitely’ would like to know the midwife attending the birth better were less likely to be satisfied with labour and birth care overall than those who did report this (caseload care OR 0.49, 95 % CI 0.32, 0.74; standard care OR 0.34, 95 % CI 0.24, 0.49).

## Discussion

In this large randomised controlled trial with high response rates at two months postpartum, women allocated to caseload midwifery care had higher satisfaction ratings for all aspects of care. These findings are in keeping with many of the RCTs [4–6, 14], other studies [4, 6, 8–14], and the Cochrane review of midwife-led care [7]. The ratings of care reported by women allocated to caseload midwifery were generally higher than those we have reported in RCTs of team midwifery care in similar populations [4, 6].

Many of the studies included in the Cochrane review did not identify what aspects of care increased women’s satisfaction [7]. In this trial, we evaluated a ‘package’ of care, and cannot draw conclusions about which specific aspects of care contributed to women’s increased satisfaction outcomes, given there were a number of aspects of care likely to have been different, including known care provider, shorter clinic waiting times, continuous support in labour, and more postnatal home visits. We did not seek to identify any one measure associated with satisfaction, but have presented comprehensive data on a range of aspects of care that together have contributed to women’s increased overall satisfaction, as well as data on individual factors associated with increased satisfaction.

In terms of exploring if continuity of carer increases women’s satisfaction, it may be that it is individual providers’ approaches to care rather than a known care provider that lead to increased satisfaction, as suggested by Green et al [15]. In this study, as in all trials in the Cochrane review of midwife-led care, midwives self-selected to caseload and may have differed in some ways to standard care midwives; the caseload midwives might have had different personal attributes or philosophies of care. The secondary analyses we presented here on continuity of carer contribute to the debate, however these analyses were not conducted by intention to treat, so the results should be interpreted with caution. In the standard care group there was no association between knowing the intrapartum care provider and satisfaction with care, however this may be due to small numbers; only 9.0 % of women in standard care reported having previously met a midwife who provided labour care. We also explored the ‘dose response’ to continuity of carer during labour and birth in the caseload care group. The sample size in each category was inadequate to provide sufficient statistical power for comparisons, however there was a suggestion that the more times a woman has previously met a midwife providing labour care (compared to not having met any of the midwives), the higher the overall satisfaction with care, up to a point where the woman has met one of the midwives four times previously. After this point there appeared to be no increase in satisfaction; however the study is underpowered to reach a conclusion on this.

On balance it seems reasonable to conclude that continuity of carer helps in part to explain the increased satisfaction in the caseload arm; however group allocation had a stronger effect than having a known care provider in the analysis we presented on overall rating of labour and birth care. Other factors are also likely to have an impact, for example women’s views and experiences of any interventions, and caseload midwives’ personal attributes [2].

The study findings need to be interpreted in context. This was a single site trial, including English-speaking women who were low risk at the time of pregnancy booking. The women in the study were more likely to be married or living with a partner or expecting their first baby and less likely to be born in Australia than the overall Victorian population [22, 23]. The survey responders were also slightly less likely to have a low family income and to be receiving government benefits as the main family income than the trial sample overall, and slightly more likely to be born in Australia and to have English as a first language.

## Conclusions

For women at low risk of medical complications, caseload midwifery increases women’s satisfaction with antenatal, intrapartum and postpartum care. Further work could explore the complex issue of the ‘dose’ of continuity of carer that is required to affect women’s satisfaction with care, at the same time ensuring the sustainability of the model from the workforce (midwife) perspective.

## Abbreviations

COSMOS: COmparing Standard Maternity care with One to one midwifery Support
GP: general medical practitioner
NHMRC: National Health and Medical Research Council
RCT: randomised controlled trial
the Women’s: Royal Women’s Hospital (Melbourne)
